# AnvRV virus in the parasitoid wasp *Anagyrus vladimiri*: localization, effect on gene expression, and prevalence

**DOI:** 10.1128/spectrum.01636-25

**Published:** 2026-05-26

**Authors:** Gal Wodowski, Yehuda Izraeli, Netta Mozes-Daube, Gon Carmi, Elad Chiel, Einat Zchori-Fein

**Affiliations:** 1 Department of Entomology, Newe Ya'ar Research Center, ARO - Volcani Institute, Ramat Yishai, Israel; 2 Department of Evolutionary and Environmental Biology, University of Haifa 411386, Haifa, Israel; 3 Shamir Research Institute, University of Haifa, Kazerin, Israel; 4 Bioinformatics Unit, Institute of Plant Sciences, Newe Ya'ar Research Center, ARO - Volcani Institute, Ramat Yishay, Israel; Barnard College, New York, New York, USA

**Keywords:** mealybugs, transmission electron microscopy, virus transmission, transcriptome

## Abstract

Insect-virus associations have been studied extensively in the context of pathogenic viruses transmitted by insects, whereas research on nonpathogenic viruses remains relatively scarce. Recently, we discovered three nonpathogenic RNA viruses in the parasitoid wasp *Anagyrus vladimiri*: AnvRV, AnvDV, and AnvIfV. Here, using transmission electron microscopy, we detected AnvRV in the wasp oocytes and in a distinct group of cells in the ovaries, which we termed “satellite cells,” but not in the venom gland or venom reservoir, indicating that AnvRV is transmitted transovarially. Next, we analyzed gene expression in AnvRV-infected and uninfected wasps and found that AnvRV modulates the immune response and alters venom composition. Notably, the NF-κB inhibitor gene was upregulated in the wasp ovaries, where AnvRV is localized, suggesting that AnvRV locally suppresses the immune response of *A. vladimiri* to facilitate its transmission. Next, we studied the prevalence of the three viruses in field populations of *A. vladimiri* and its hosts, *Planococcus citri* and *Planococcus ficus*. AnvRV was absent from both mealybug species and detected at low prevalence in *A. vladimiri*, whereas AnvDV and AnvIfV were consistently present in *P. citri*. Lastly, by datamining of public RNAseq data sets, we investigated the prevalence of these viruses in other parasitoid species and revealed only a few related viruses. Taken together, we postulate that AnvRV is an active symbiont of *A. vladimiri*, influencing host gene regulation. These findings provide new insights into the diversity of insect–virus interactions and their potential roles in shaping parasitoid biology.

**IMPORTANCE:**

Viruses likely represent the majority of insect symbiotic microorganisms. Yet, viral symbionts and their interactions with insect hosts were less studied, mostly due to technical difficulties stemming from their small size and lack of universal markers. Although viral symbionts are usually perceived as pathogens, there are clear instances in which they are beneficial to their hosts, providing functions that are essential in some cases and conditionally beneficial in others, shaping insect ecology and evolution. This study provides several pieces of the puzzle on the road to understanding the complex interactions within the multi-trophic system consisting of a parasitoid wasp, its mealybug host, and a double-stranded RNA virus. This system may serve as a case study of viruses’ effect on insects and broaden our understanding of the possible effects of viruses on other arthropods.

## INTRODUCTION

Eukaryotes are both heterogeneous (made of entities of different origins, often belonging to different kingdoms) and heteronomous (depend on other biological entities to complete their life cycle) (1). Symbiotic interactions, including mutualism, parasitism, and commensalism, between microorganisms and eukaryotes are widely recognized for creating life as we know it, enabling diet expansions, inhabitation of unexploited niches, and species diversification (2).

Viruses likely represent the majority of insect symbiotic microorganisms (3), yet, unlike bacterial and fungal symbionts, viral symbionts and their interactions with insect hosts were less studied, mostly due to technical difficulties stemming from their small size and lack of universal markers. Although viral symbionts are usually perceived as pathogens, there are clear instances in which they are beneficial to their hosts, providing functions that are essential in some cases and conditionally beneficial in others, shaping insect ecology and evolution (4–6).

The model system in the current study is a virus in a parasitoid. Parasitoids are insects (the vast majority are wasps and flies) that feed and develop on or inside the body of another arthropod host while the adults are free-living. Parasitoid wasps seem to be highly inclined to long-term relationships with viruses (7, 8). One of the most studied groups of mutualistic insect viruses is the polydnaviruses (PDVs), which are dsDNA viruses associated with parasitoid wasps of the families Braconidae and Ichneumonidae. PDVs suppress the immune system of the wasps’ hosts “lepidopteran caterpillars“ thus enhancing the successful development of the wasp larvae. PDVs are endogenous, i.e., their genomes have been completely integrated into the wasps’ genome and thus their definition as symbionts, i.e., a separate entity from the host, is debated (9). However, there are plenty of reported cases of non-endogenous viral symbionts of insects (5, 10). For example, an entomopoxvirus (dsDNA, Poxviridae) symbiont of a braconid parasitoid wasp is injected into tephritid fly hosts and suppresses host immunity (11, 12). Another braconid parasitoid wasp species injects an ssRNA virus (Iflaviridae) into its ladybird host, the virus then replicates in the ladybird’s brain and renders it a “zombie” that guards the parasitoid cocoon from predation (13). Conversely, some aphid species are protected from parasitoids by mutualistic Podovirus-like bacteriophages, which live in facultative bacterial symbionts of the aphids and encode toxins that eliminate parasitoid eggs (14).

The focus of the current study is the parasitoid wasp *Anagyrus vladimiri* Triapitsyn (Hymenoptera: Encyrtidae), a natural enemy of several mealybug species (Hemiptera: Pseudococcidae) (15), that is used as a biocontrol agent of global pest mealybug species. *Anagyrus vladimiri* is a solitary koinobiont endoparasitoid, i.e., female wasps oviposit one or more eggs into the mealybug’s body, the hatched larvae feed on the internal organs of the mealybug host (which dies after a few days), pupate inside the dead host cadaver (termed mummy), and eventually emerge from the mummified mealybug bodies (16). “Solitary” means that only one offspring completes its development from each host, even if more than one egg was laid (superparasitism). “Koinobiont” means that the mealybug host is not killed or paralyzed by the wasp during oviposition, but the parasitized mealybug continues to live until the wasp larva has consumed most of its internal organs (17).

Some of the wasp eggs are eliminated by the mealybug’s immune response, which involves hemocytes that encapsulate the egg and then release melanin and reactive molecules (18). To counter these defenses, koinobiont parasitoids employ virulent factors that suppress the host’s immune response and regulate host metabolism. Given these immune challenges, superparasitism is believed to be a wasp strategy to increase the chance that at least one egg will successfully hatch (19).

In a previous study, we reported the finding of three symbiotic viruses of *A. vladimiri* from three different families: Reoviridae, Iflaviridae, and Dicistroviridae (AnvRV, AnvIfV, AnvDV, respectively) (20). The two latter viruses were found in unparasitized mealybugs, while AnvRV is associated with the wasp and not with the mealybug host. AnvRV, like other reoviruses, has a segmented linear dsRNA genome comprised of 10 segments ranging from 1.5 to 4.2 kb in length, but only two out of the 10 segments, the RDRP and a capsid protein, have been annotated (20).

In a more recent study, we found that AnvRV is transmitted maternally with very high fidelity across multiple generations and can also be horizontally transferred from infected to uninfected wasp larvae developing within the same mealybug host (superparasitism). The most intriguing phenotypic effect of AnvRV is that the hatching rate of eggs of AnvRV^+^ *A. vladimiri* is dramatically higher than those of AnvRV^−^ (21).

The present study focuses on various aspects of the interaction between AnvRV, AnvDV, and AnvIfV and the adult *A. vladimiri,* and the ecological context of these viruses. First, to elucidate if AnvRV is transmitted transovarially or/and via fluids that are injected with the eggs, the localization of AnvRV in various *A. vladimiri* tissues was mapped using transmission electron microscopy (TEM) and molecular tools. Second, to gain more knowledge of AnvRV*–A. vladimiri* interaction, a comparative RNAseq, as well as qPCR analyses were applied, comparing AnvRV^+^ and AnvRV^−^ adult wasps. Third, the prevalence of the three viruses in parasitoids and mealybugs collected in the field was assessed, and finally, a broad survey was conducted, searching for closely related viral sequences in other Chalcidoid wasps and mealybugs using data mining.

## RESULTS

### Anatomy of *A. vladimiri* reproductive system

**Fig 1:**
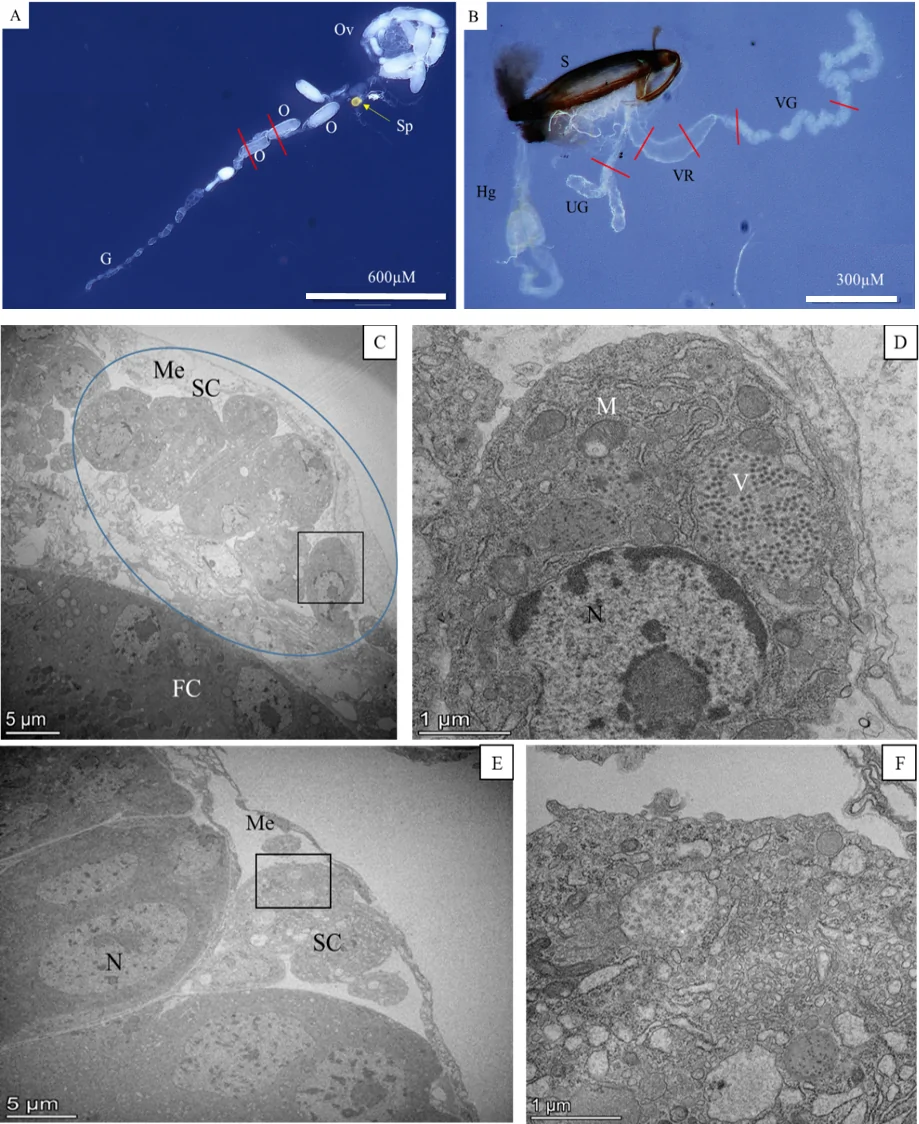
Reproductive tract of *A. vladimiri* female. (**A**) An ovary (Ov) with one ovariole spread out (from the top right corner to the bottom left corner). The red lines represent the sectioning locations for TEM: Sp, Spermatheca; O, Oocyte; G, germarium. (**B**) Maternal injection system; VG, venom gland; VR, venom reservoir; UG, unidentified gland; Hg, hindgut; S, Sheath. (**C–F**) TEM of *A. vladimiri* ovaries. (**C**) “Satellite” cells (SC, encircled by the blue ellipse) next to a follicle cell (FC), within the ovarian membrane (Me). The area marked with a square is shown in higher magnification in Fig. 2D. (**D**) A satellite cell showing a membrane-enclosed structure full of viruses (V). (**E**) Satellite cells of AnvRV^−^ *A. vladimiri*. M, mitochondrion; N, nucleus. (**F**) Enlargement of “E” in the black square, no virus structures found in the cells.

Dissections of *A. vladimiri* adult females revealed a few distinct components, including (i) the ovaries, which are generally typical of other species of parasitoid wasps, with polytrophic meroistic ovarioles (i.e., the nurse cells are located near the oocyte, Fig. 1A); (ii) the maternal injection system which consists of the venom gland, venom reservoir and another unidentified gland (possibly Dufour’s gland, Fig. 1B). The anatomy of these organs is highly variable in parasitoid wasps; therefore, it is challenging to classify them.

**Fig 2:**
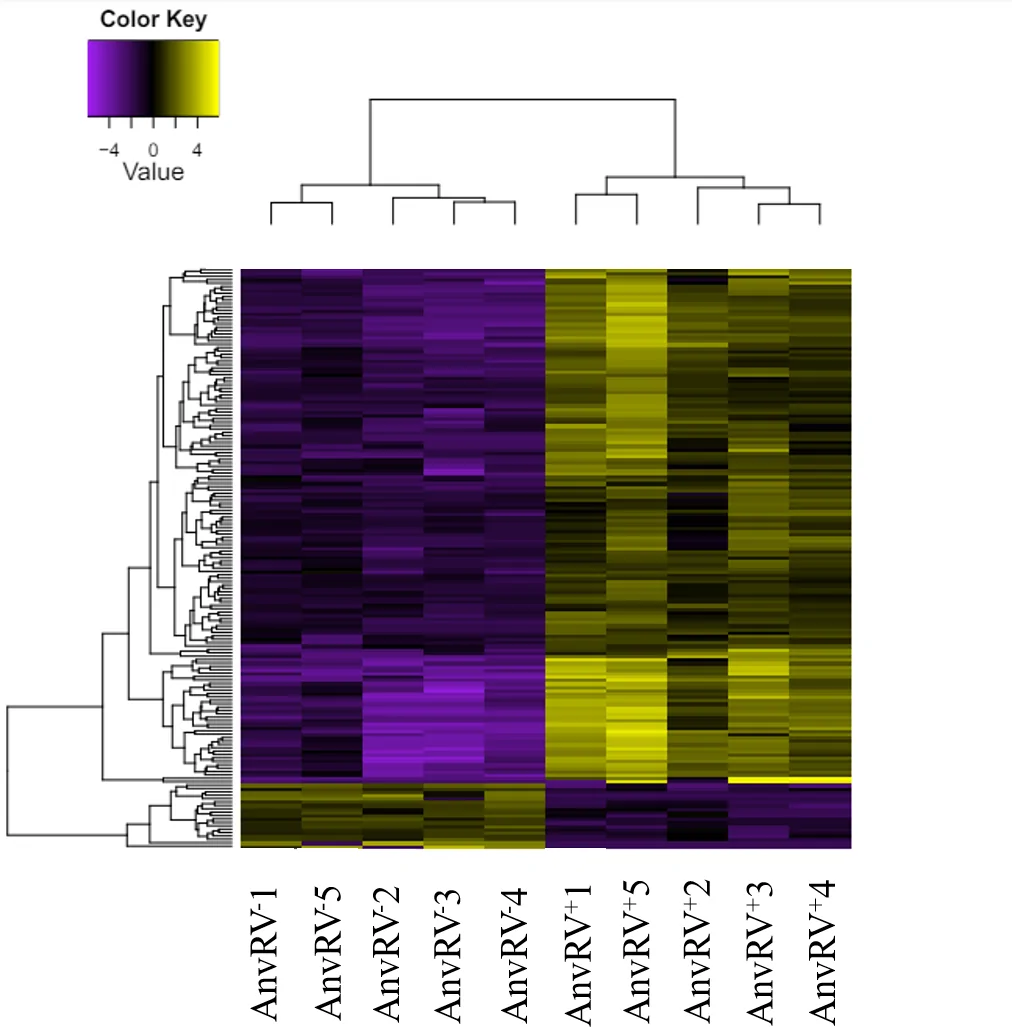
Heatmap showing transcripts (grouped by hierarchical clustering) that were differentially expressed in AnvRV^+^ and AnvRV^−^ *A. vladimiri*. Each row is a transcript, each column is a sample. Yellow represents upregulated transcripts; purple represents downregulated transcripts, and black is neutral.

### Virus localization using transmission electron microscopy

Images of the ovaries revealed large numbers of scattered AnvRV particles in a group of unidentified cells localized outside the follicle cell layer, yet within the membrane enveloping the ovarioles (hereafter referred to as “satellite cells”; Fig. 1C and D). The satellite cells could also be identified in the ovaries of AnvRV^−^ wasps (Fig. 1E and F), suggesting that the virus is “hijacking” them for its own purpose rather than causing cell proliferation. A more sporadic distribution of virus particles, but in a more geometric order, was detected in the nurse cells (Fig. S1). Conversely, when looking at the maternal injection system, no virus particles could be found in the various parts screened along both the venom gland and venom reservoir, as well as in the unidentified gland (Fig. S2).

The qPCR amplification used to further explore the localization of AnvRV in *A. vladimiri* revealed the presence of the virus in both the ovaries and in the maternal injection system, with no significant difference between the tissues (wilcoxon rank-sum test: *n* = 5 of each tissue, *w = 9*, *P* value = 0.88).

### *Anagyrus vladimiri*’s possible integration to the genome

To determine if the identified viruses are integrated into *A. vladimiri* genome, we performed PCR on genomic DNA (gDNA) extracted from AnvRV^+^ (*n* = 10) and AnvRV^−^ (*n* = 10) wasps. No amplification was recorded for AnvRV, AnvDV, or AnvIfV in any of the gDNA samples. In contrast, RT-PCR performed on RNA extracts from the same individuals yielded positive results for AnvDV and AnvIfV in all samples, while AnvRV was detected exclusively in the infected lines. The successful amplification of the host 18S rRNA gene from gDNA templates served as a positive control for DNA quality. These results indicate that AnvRV, AnvDV, and AnvIfV are exogenous RNA viruses and are not integrated within the host genome.

### Determination of differential gene expression by transcriptome analysis

The RNAseq of *A. vladimiri* yielded 23.9 and 37.8 million high-quality raw paired-end reads (>33 Phred score) in AnvRV^+^ and AnvRV^−^, respectively. The raw data are available on the SRA NCBI database (Bioproject accession PRJNA1033425). *De novo* assembly yielded 72,170 transcripts, with a median and average transcript lengths of 468 and 1,258 bp, respectively. One hundred seventy-eight transcripts were upregulated, and 102 were downregulated in AnvRV^+^ compared to AnvRV^−^ *A. vladimiri* (Fig. 2; Supplemental material), 76 of which were of unknown biological function. Out of the 280 differentially expressed (DE) transcripts, 73 transcripts were found in the venom of hymenopterans’ proteome or transcriptome (Supplemental material).

**Fig 3:**
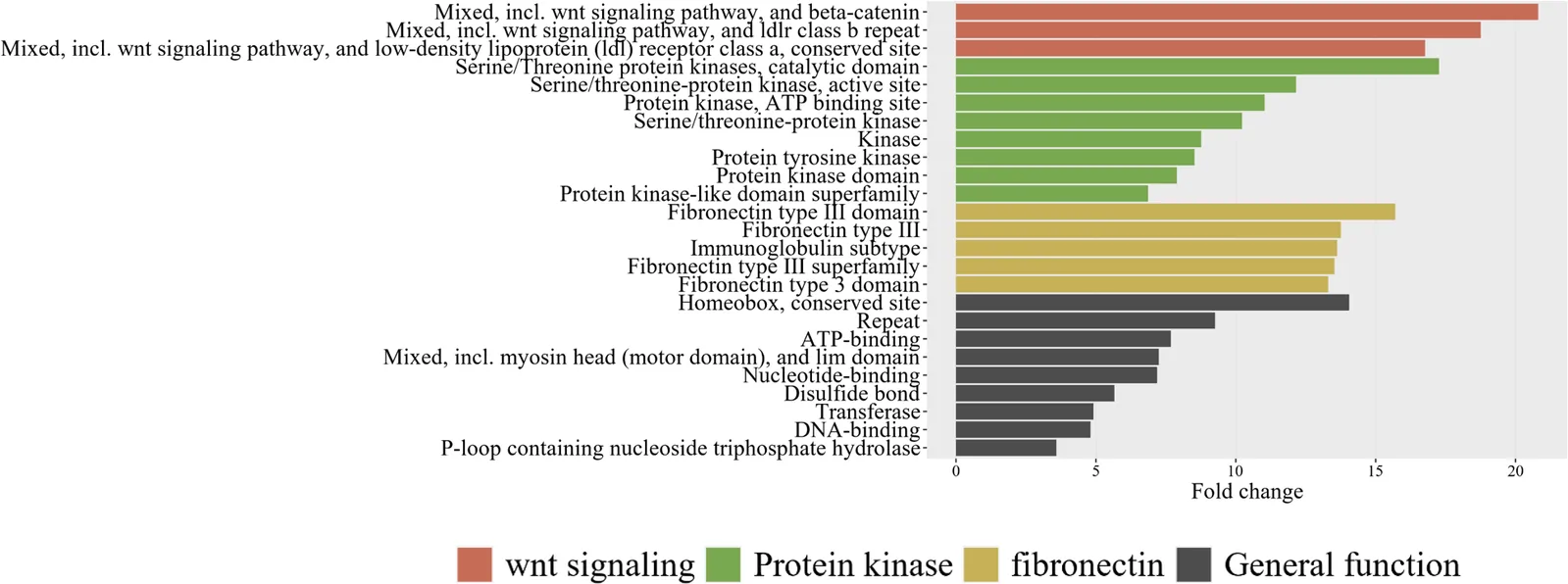
Gene Set Enrichment Analysis (GSEA) of the 25 most differentially expressed pathways. Colors indicate different biological functions. Y-axis, gene family; X-axis, total fold change of gene sets, both upregulated and downregulated genes in the gene set. All annotations were used by Gene Ontology terms.

The gene set enrichment analysis (GSEA) resulted in 319 DE (enrichment FDR < 0.01) gene sets (see Supplemental material). Out of the 25 most significantly DE gene sets (with the lowest FDR values), three were associated with the Wnt signaling pathway, eight sets were protein kinases (mostly serine or tyrosine kinases), and four sets were of fibronectin (Fig. 3).

### Quantification of selected *Anagyrus vladimiri* genes by qPCR

**Fig 4:**
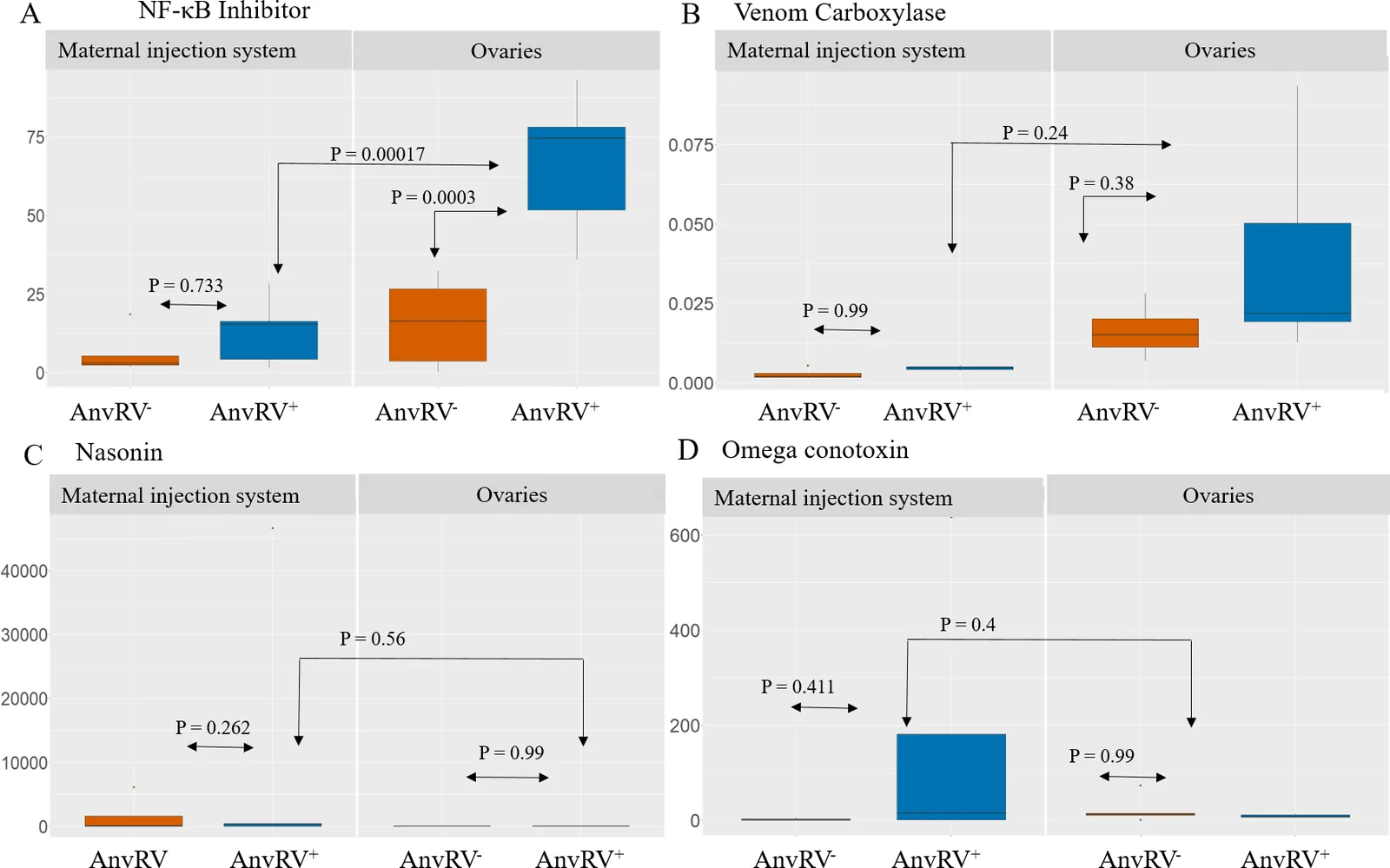
Expression levels of the four selected genes in the ovaries and in the maternal injection system of AnvRV^−^ and AnvRV^+^ wasps. (**A**) NfkB inhibitor; (**B**) Venom carboxylase; (**C**) Nasonin; (**D**) Omega conotoxin. Orange bars represent AnvRV^−^ wasps and blue bars represent AnvRV^+^ wasps.

As mentioned in the Methods and Materials, four genes that were differentially expressed between the ovaries and the maternal injection system were selected for further analysis by qPCR: Omega conotoxin, Nasonin, NF-κB inhibitor, and venom carboxylase. NF-κB inhibitor expression was found to be significantly higher in the ovaries of AnvRV^+^ vs AnvRV^−^ wasps (Fig. 4A), and within AnvRV^+^ wasps, it was much higher in the ovaries compared to the maternal injection system. Carboxylase was downregulated in both tissues of AnvRV^+^ (note the Y-axis values), and its expression was lower but not significant in the maternal injection system compared to the ovaries in both wasp lines (Fig. 4B). Nasonin and Omega conotoxin expression was not significantly different between the organs and lines (Fig. 4C and D).

### A screening of *Anagyrus vladimiri* viruses by data mining

A total of five sequences of RNA viruses were found in the SRA data sets screened, three of which were previously undescribed. The viruses were detected in wasps from different families and various hosts. AnvDV sequences were found in *Leptomastix dactylopii* (Encyrtidae), a parasitoid wasp of mealybugs; *Diversinervus elegans* (Encyrtidae), a parasitoid wasp of soft scales, and *Pteromalus puparum* (Pteromalidae), a parasitoid wasp of butterflies in their pupal stage (Table 1). The AnvIfV sequences were found in *Metaphycus flavus* (Encyrtidae), a parasitoid wasp of soft scales, and in *Phymastichus coffea* (Eulophidae), a parasitoid wasp of coffee beetles (Table 1). No AnvRV sequences were found (excluding the positive controls originated from the Mexican vine mealybugs).

**TABLE 1:** Screening for *A. vladimiri* viruses via data-mining; the 6 SRA data sets that were found positive, out of 83 chalcidoid data sets that were surveyed^*c*^^,^^*d*^^,^^*e*^

SRA study	Species	Family	Parasitized host	AnvRV	AnvIfV	AnvDV	Assembled contig (TSA database)
SRR19049041 ^*a*^	*Anagyrus vladimiri*	Encyrtidae	Mealybugs	+	+	+	OP349052–OP349063
SRR5369853	*Diversinervus elegans*	Encyrtidae	Scale insects	−	−	+	MT127560.1
SRR1503005	*Leptomastix dactylopii*	Encyrtidae	Mealybugs	−	−	+	GBNE01004622.1
**SRR1502934** ^*b*^	***Metaphycus flavus***	**Encyrtidae**	**Soft scale insects**	−	**+**	−	**GBVC01018549.1**
**SRR1823062/SRR1823064**	***Pteromalus puparum***	**Pteromalidae**	**Butterflies**	−	−	**+**	**GECT01016007.1**
**SRR25496109**	***Phymastichus coffea***	**Eulophidae**	**Beetles**	−	**+**	−	**This study**
SRR16213284 (positive control, mealybugs from Mexico)	*Planococcus ficus*	Pseudococcidae [Mealybug]	N.A.	+	+	+	

^*a*^ SRA from our previous study.

^*b*^ Found via DIAMOND_BLAST in a preliminary SRA screening.

^*c*^ Bold indicates novel viruses that were discovered in this study.

^*d*^ −, not found; +, found.

^*e*^ N.A., not available (not a parasitoid).

**Fig 5:**
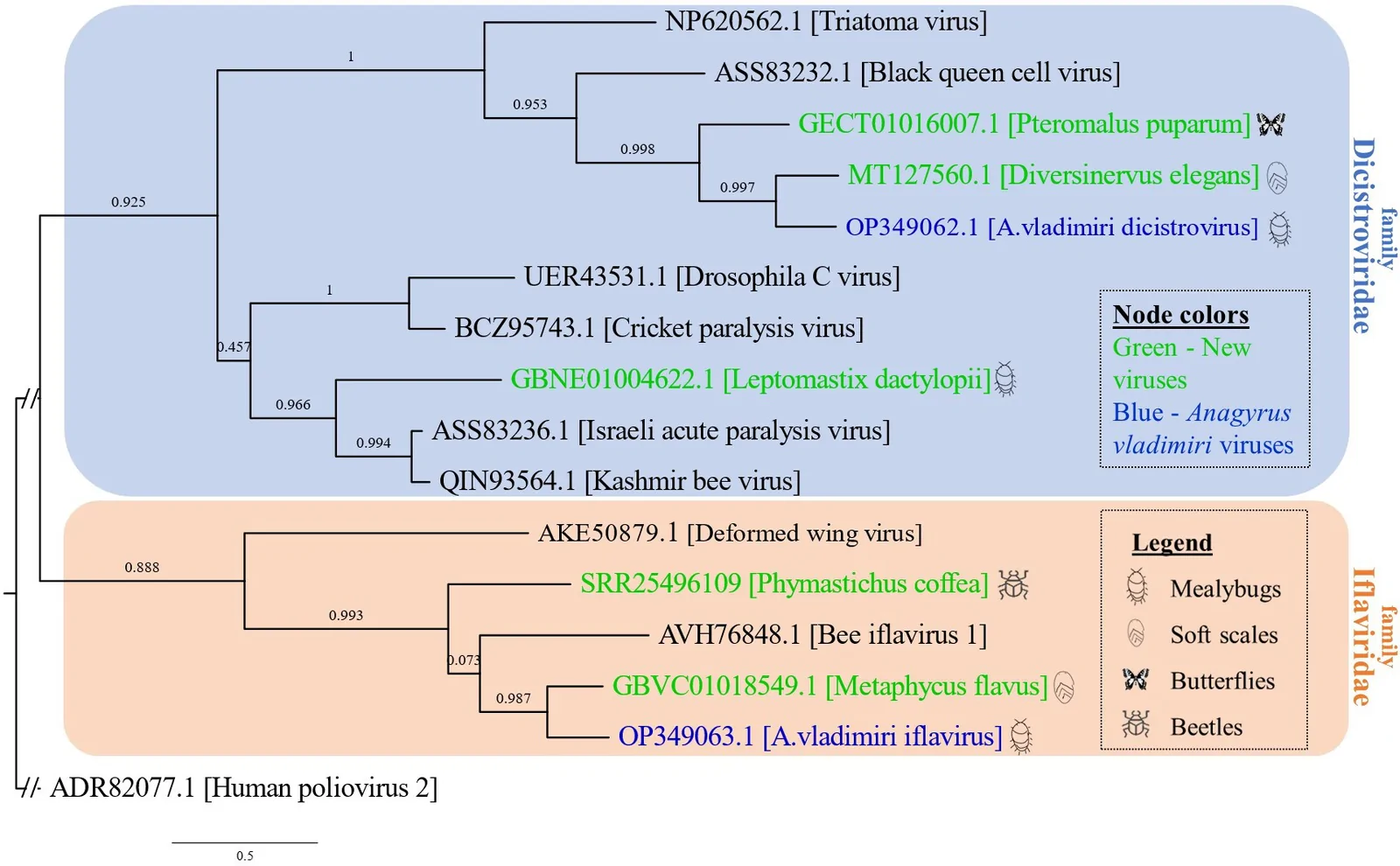
Phylogram of the new viruses found in the SRA screening, based on amino acid sequence similarities of the RdRP coding gene (~300aa long). Newly discovered viruses are written in green font, *A. vladimiri* viruses are colored blue. The icons of organisms on the tips represent the insect order parasitized by the wasp’s species in which the viruses were found. The human poliovirus (order: Picornavirales, family: Picornaviridae) was used as an outgroup. The tree was inferred by maximum-likelihood, and numbers on branches indicate the results of approximate likelihood ratio tests (branch support, aLRT-SH). Scale bar, 0.5 substitutions per site.

The phylogenetic analysis of the relationships between the newly discovered viruses and the *A. vladimiri* viruses revealed a non-concentrated pattern; two out of three of the AnvDV-related viruses clustered on the same clade with AnvDV, while the third (found in *L. dactylopii*) belongs to a different genus within the Dicistroviridae family. Similarly, the AnvIfV-related viruses also clustered on different branches (Fig. 5).

The phylogeny of the new viruses did not reflect the phylogeny of their wasp hosts. For example, the Dicistrovirus found in *P. puparum*, family Pteromalidae, was closer to AnvDV than the Dicistrovirus found in *L. dactylopii,* an encyrtid wasp which, like *A. vladimiri,* attacks mealybugs. This suggests that the genomes of these viruses are not strictly conserved, and some horizontal transfer between species possibly occurs.

Compared to the five viruses discovered in the chalcidoid wasps, the screening of the data sets originated from mealybugs revealed not even one viral sequence in any of the 134 SRAs screened (excluding the positive controls) (RNA datamining table).

### Prevalence of the viruses in field-collected wasps and mealybugs

As expected, both molecular analyses and morphological examination identified the specimens collected from vines and citrus as the vine mealybug *P. ficus* and the citrus mealybug *P. citri,* respectively. The most prevalent parasitoid in both collections was *A. vladimiri*. From *P. ficus,* several individuals of the genus *Marietta* emerged, which are known to be hyperparasitoids (i.e., parasitoids of primary parasitoid species, such as *A. vladimiri*).

AnvDV and AnvIfV were found in all the *P. citri* individuals (collected from citrus), but in none of the *P. ficus* individuals (collected from vines). As expected, AnvRV was not found in any unparasitized mealybug. Among parasitoids that emerged from *P. ficus,* AnvRV was found in ~15% of *A. vladimiri* and ~55% of *Marietta* spp. Similarly, AnvRV prevalence in *A. vladimiri* that emerged from *P. citri* was ~10%. AnvDV and AnvIfV were not found in parasitoids that emerged from *P. ficus*, while in parasitoids that emerged from *P. citri,* their prevalence was 10% and 30%, respectively (Table 2).

**TABLE 2:** Prevalence of AnvRV, AnvDV, and AnvIfV in field collections of mealybugs and their parasitoids^*a*^

		Parasitoids		Mealybugs	
	Virus	*Anagyrus vladimiri*	*Marietta* spp.	*Planococcus ficus*	*Planococcus citri*
Collected from vines	AnvRV	*5/33*	*7/13*	0/10	–
	AnvDV	0/13	0/11	0/10	–
	AnvIfV	0/13	0/11	0/10	–
Collected from citrus	AnvRV	*2/23*	–	–	**0/10**
	AnvDV	*4/23*	–	–	**10/10**
	AnvIfV	*6/23*	–	–	**10/10**

^*a*^ Bold numbers indicate full prevalence; Italicized numbers indicate partial prevalence; –, not found.

## DISCUSSION

The main findings of the current study are (i) the vertical transmission of AnvRV is transovarial; viral particles were seen inside oocytes, in the surrounding nurse cells, and attached to the ovarioles, but not in the venom gland or venom reservoir (Fig. 1/S1-2). (ii) Transcriptomic analysis revealed differential gene expression between AnvRV^+^ and AnvRV^−^ wasps, while targeted qPCR validation identified tissue-specific expression changes in the ovaries (Fig. 4). (iii) AnvRV is a symbiont of the parasitoid wasp *A. vladimiri*, whereas AnvDV and AnvIfV are symbionts of *P. citri*. (iv) AnvRV is not integrated into the wasp genome. Also, none of the three viruses was found in *P. ficus* (Table 2). (v) The presence of newly characterized “satellite cells” surrounding follicle cells in the ovariole has the potential to broaden our understanding of parasitoid anatomy. (vi) The discovery of three novel RNA viruses inhabiting three different economically important parasitoids from diverse taxonomic groups (Table 1).

### Virus transmission pathways

The transmission of reoviruses in parasitoid wasps can occur through various mechanisms (reviewed by Renault [17]). *Hyposoter exiguae* idnoreovirus-2 is transmitted during oviposition and replicates in multiple wasp tissues (22). Reoviruses like *Diadromus pulchellus* reovirus (DpRV-2) and *Operophtera brumata* reovirus are also transmitted during oviposition (23, 24), with DpRV-2 primarily replicating in host midgut cells. *Diadromus pulchellus* idnoreovirus-1 is found in various wasp gut tissues and venom gland, and is released into the gut lumen and is transmitted to the Lepidopteran host during oviposition, but does not replicate there. However, information regarding the precise localization of these viruses within their hosts remains incomplete or poorly documented.

Recently, we reported that AnvRV vertical maternal transmission rate is nearly 100%, while horizontal transmission occurs via superparasitism with ~25% efficiency (21). Additionally, we documented the presence of AnvRV inside the oocyte (20), strongly suggesting a transovarial transmission. Here, AnvRV was found again in the oocytes, corroborating our previous findings, as well as in the nurse cells in an organized and structured way (Fig. S1). Interestingly, we detected unidentified cells outside the follicle cell layer yet inside a membrane, possibly the ovariole membrane, which all have an organelle-like structure containing AnvRV (Fig. 1C and D). The structure of the organelles inside the cell and the virus particles inside the organelles are very similar to the viral factories of other reoviruses (25, 26). These tentatively termed “satellite cells” were also found in AnvRV^−^ *A. vladimiri* (but lacked virus particles; Fig. 1E and F), excluding the option that they are virus-specific cells. While they reside adjacent to mature oocytes, they remain distinct from follicle cells as they do not encircle the oocyte. Furthermore, their location differs from that of typical nurse cells, which are generally associated with younger oocytes. Although these may represent nurse cells captured in a non-representative two-dimensional plane, their function remains unknown. AnvRV may utilize these cells for replication to facilitate subsequent inoculation of the oocytes.

Using TEM, AnvRV could not be detected in the maternal injection system in several dissections across the glands and reservoir (Fig. 1B; Fig. S1), unlike several other reoviruses in parasitoid wasps (17). In contrast, using qPCR analysis, we did detect AnvRV in the maternal injection system, suggesting potential viral presence in the venom gland and/or reservoir as well. This discrepancy may stem from a low viral abundance that eluded detection during histological sectioning. Alternatively (and more likely, in our opinion), the qPCR signal could reflect circulating AnvRV particles in the hemolymph or contamination from other tissues that were damaged during the dissections, rather than localization within the tissue itself. Therefore, currently, it cannot be definitively determined whether AnvRV is injected into the host via the maternal fluids. Both possibilities, therefore, remain open.

The finding of AnvRV in *Marietta* sp. developing in the same mealybug host population (Table 2) suggests that the physical contact between *A. vladimiri* and the hyper-parasitoid might be the cause of AnvRV transmission, resembling AnvRV horizontal transmission occurring during superparasitism (21). Future studies should investigate whether AnvRV replicates within *Marietta* spp. tissues and whether AnvRV can be vertically transmitted.

### AnvRV potential involvement in immunity

Our hypothesis was that the higher hatching rate of AnvRV^+^ eggs compared to AnvRV^−^ eggs (21) is due to an AnvRV-induced inhibition of the mealybug’s immune response. This hypothesis is only partially supported by our data. Some DE pathways are correlated with immune response or venom alterations, though their exact roles remain to be fully elucidated. For example, WNT/beta-catenin, a conserved pathway governing cellular proliferation, development, and the self-renewal of cells, was reported by several studies to be manipulated by viruses in a way that will promote infection (27, 28). Conversely, Li et al. (29) found that the WNT pathway is important in the immune response of *Samia ricini* (Lepidoptera: Saturniidae) against a pathogenic baculovirus.

Another intriguing example is the protein kinases, which are known to be involved in many processes, including, on one hand, the immune response, mainly the activation of the melanization process by serine protease (18, 30), and on the other hand, may be part of the parasitoid wasps’ venom protein repertoire (31). Here again, upregulation of protein kinases can be explained by *A. vladimiri’s* immune response to AnvRV, as well as hint at possible changes in venom composition that will benefit the fitness of the wasp. Since in this study the RNA sequences are from whole wasps, the specific location of the changes cannot be determined, and both options remain plausible.

To address this issue, we conducted a qPCR analysis of specific genes found to be DE in the transcriptome analysis in two different tissues, the ovaries and the maternal injection system (Fig. 1A and B). Based on the TEM images, showing viral particles in the ovaries (Fig. 1) but not in the venom gland, a stronger immune response was expected where the viruses were seen. NF-κB Inhibitor was upregulated in AnvRV^+^ ovaries compared to AnvRV^−^ ovaries (Fig. 5A), suggesting that the virus inhibits the immune response of *A. vladimiri*, which can explain the lack of clear immune-related pathways in the GSEA analysis, e.g., Toll, Imd, and JNK kinase (Fig. 3).

The GSEA results, together with the qPCR results, suggest a local inhibition of the immune response in the wasp ovaries. Future studies can expand to more genes in the immune system and in more tissues (mainly gut, fat body, and brain).

Parasitoid wasp venom is a complex and highly variable mixture of proteins, even among closely related species (32). In this study, 70 out of the 73 annotated DE transcripts, potentially associated with venom (Supplemental material), were upregulated in AnvRV*^+^* wasps compared to AnvRV*^−^* wasps. This result suggests that AnvRV influences the venom composition of *A. vladimiri*, either as a direct manipulation, such as viral interference with host transcriptional regulation, or viral proteins modulating venom gland function, or as a host response to the virus. Many DE genes identified are multifunctional; they were found in the venom of other parasitoid wasps and might potentially be part of *A. vladimiri* venom, and also might be part of other processes (e.g., metabolism or immune response). Some of the venom-associated DE genes encode toxins (e.g., Omega conotoxin), which are likely integral components of the venom.

Both Omega conotoxin and Nasonin were upregulated in AnvRV^+^ wasps (Supplemental material) and most likely are part of the venom fluid; however, no differences in the expression levels of these genes could be identified in the ovaries or the maternal injection system between AnvRV^+^ and AnvRV^−^ (Fig. 4). Carboxylase is a metabolic gene involved in fatty acids' synthesis, and in the venom of parasitoid wasps, it also plays a role in regulating host metabolism (33). In our analysis, gene expression was lower than the reference gene in both tissues (Fig. 4C). This suggests that carboxylase is likely not a venom-specific gene but instead a component of the wasp’s metabolism, potentially differentially expressed due to metabolic changes influenced by AnvRV infection.

In our previous work (21), we found that AnvRV*^+^* wasp eggs had a higher hatching rate than AnvRV*^−^* eggs, but there was no difference in the number of offspring between the two wasp lines. Our current results, suggesting venom composition alterations, support an alternative hypothesis: the virus does not directly affect the host’s immune response but modifies *A. vladimiri’s* venom, increasing its potency. This enhanced venom potency could be a double-edged sword. While it may facilitate egg hatching, it might also negatively impact parasitoid larval growth by reducing host nutritional value. Further research, including a comparison of venom proteome and quantity, is required to test this hypothesis.

### Virus specificity

The fact that no closely related viruses to AnvRV were found in the screening, and not even in other representative species in the family Encyrtidae, strongly supports our hypothesis that this virus is highly specific to its insect host, as has been previously suggested for insect viruses (34). This finding suggests that *A. vladimiri* and AnvRV interact in an evolutionary trajectory that can lead to a more obligate symbiosis. This is similar to what has been shown for mutualistic DNA viruses (35). On the other hand, it can lead to a parasitic trajectory in which AnvRV infection will reduce *A. vladimiri* fitness, as has been shown in the *Leptopilina boulardi* LbFV system (36).

The large-scale data mining survey revealed only five Chalcidoid wasp species that harbor any of the *A. vladimiri* viruses or closely related ones (Table 1). The only AnvRV-like virus (>90% amino acid identity) that was found by data mining was in the mealybug *P. ficus* collected from vines in Mexico (37). In light of our finding that AnvRV is a symbiont of *A. vladimiri* and not mealybugs, we speculate that the *P. ficus* sampled by Martinez et al. (37) were actually parasitized by *A. vladimiri*. This speculation is further supported by the finding of some reads showing 100% identity with the COI gene of *A. vladimiri* in their database (Gal Wodowski, unpublished data). This observation highlights an important consideration when interpreting virus-host associations derived from transcriptomic or metagenomic data, particularly in insect systems where parasitism is common. Our findings do not diminish the value of the original discovery but rather emphasize the complexity inherent to studying host-virus relationships in ecologically intricate systems. Similarly, our data mining analysis found AnvDV and AnvIfV only in chlacidoid wasps, but we cannot rule out the possibility that the virus’s origin is in fact their insect host.

The prevalence of the viruses in field-collected wasps and mealybugs supports the previous report claiming that AnvDV and AnvIfV are mealybug viruses. However, in the datamining results, closely related viruses were found only in Chalcidoids and none from over 100 RNAseq studies of mealybugs. Further research is needed to conclude what is the true origin (i.e., primary host) of AnvDV and AnvIfV. It may also be possible that these two viruses are not widespread in mealybugs worldwide.

While the two encyrtid wasps, *D. elegans* and *L. dactylopii*, were already reported to carry Dicistroviruses (38, 39), the discovery of RNA viruses in *M. flavus*, *P. puparum,* and *P. coffea* is novel to the best of our knowledge.

In conclusion, this study provides several pieces of the puzzle on the road to understanding the complex interactions within the multi-trophic system consisting of a parasitoid wasp, its mealybug host, and a double-stranded RNA virus. Additional insights into viral-host specificity aspects, as well as the diverse ways by which viruses are moving within and between hosts, will allow the establishment of wasp lines with different viral compositions and might eventually lead to the development of more efficient natural enemies applied in biological control programs.

## MATERIALS AND METHODS

### Insect origin and maintenance

The study system included *P. citri* reared on sprouted potatoes, which served as hosts for two isogenic lines of the parasitoid *Anagyrus vladimiri*, one that carries AnvRV (AnvRV^+^) and one that does not (AnvRV^−^). These isogenic lines were previously established in our lab by transferring the AnvRV between siblings (for detailed description, see reference 21). All insects and experiments were maintained under controlled conditions of 26°C ± 1°C, 65% ± 15% RH, and a 16L:8D photoperiod regime.

### Anatomy of *A. vladimiri* reproductive system

In a previous work, we showed that AnvRV is vertically transmitted from mother wasps to offspring with very high fidelity (21), but only a small number of viral particles could be seen in follicle cells of *A. vladimiri* using TEM (20). Parasitoid wasps inject components that play a vital role in the parasitization process, including venom and/or ovarian/calyx fluid (40). Thus, our working assumption was that in addition to the follicle cells, AnvRV might be localized in the wasp’s venom gland, ovaries, or calyx fluids (hereafter collectively termed maternal injection system), and injected into the mealybug during parasitization. To determine the localization of AnvRV in *A. vladimiri,* it was first imperative to identify the various parts of the reproductive system. Hence, 50 AnvRV^+^ and 50 AnvRV^−^ *A. vladimiri* females were dissected in a droplet of PBS, the reproductive system was gently removed from the wasp’s body, and the ovaries and maternal injection system were separated under a stereoscopic microscope (Olympus SZX12). The various organs were documented by a 3D digital microscope (Hirox RH-2000).

### *Anagyrus vladimiri*’s possible integration to the genome

To determine if AnvRV is a “free living” virus or integrated into the genome of *A. vladimiri,* we extracted DNA and RNA from 10 infected and 10 uninfected *A. vladimiri* (DNA and RNA extracted from separate samples) using the NucleoSpin kit (Macherey Nagel). RNA samples were reverse-transcribed to cDNA using the RT-PCRbio kit (PCR Biosystems). The samples of extracted gDNA and cDNA were tested for *A. vladimiri* viruses (AnvRV, AnvDV, AnvIfV) using the same diagnostic primers as reported by Izraeli et al. (20). The logic in this test is that if AnvRV is integrated into *A. vladimiri*’s genome, then it will be amplified in both gDNA and cDNA samples, whereas if it is a “free-living” virus, it will be amplified only in cDNA samples.

### AnvRV localization using transmission electron microscopy

To determine the presence of the virus in the reproductive system, ovaries, Dufour and venom glands of AnvRV^+^ and *AnvRV^−^ A. vladimiri* females were fixed overnight with 2% paraformaldehyde, 2% glutaraldehyde in 0.1 M cacodylate buffer pH 7.4, washed in 0.1 M cacodylate, post-fixed for 1 h in 1% OsO_4_ in cacodylate buffer and en-block stained with 1% uranyl acetate for 1 h. Then the samples were dehydrated in serial ethanol solutions (50%, 70%, and 96% for 25 min each, then 100% for 45 min) and were embedded in Epon 812 (EMS). The blocks were cut with a diamond knife using a UC7 ultramicrotome (Leica). Sections of ~75 nm were collected on grids and visualized by TEM (Talos L120C, Thermo Fisher Scientific).

### Analysis of differential gene expression by transcriptome analysis

To investigate how AnvRV influences *A. vladimiri* at the molecular level, RNAseq analysis comparing the transcriptome of the isogenic wasp lines AnvRV^+^ vs AnvRV^−^ was conducted (for a detailed description of the establishment of these two lines, see reference 21). The wasps used for this analysis were ~50 generations post the establishment of the two isogenic lines. Genetic drift is possible after 50 generations; however, in this case, it is less likely as both lines are maintained under the same conditions, and the populations are large enough (>50 wasps every generation) to avoid genetic bottlenecks. RNA was extracted from virgin adult female wasps 0–24 h post-emergence (*n* = 5 for each line) using the NucleoSpin RNA kit (Macherey-Nagel) according to the manufacturer’s manual. RNA integrity was assessed using Tapestation Agilent Technologies (41). The samples were sequenced by Genewiz (South Plainfield, NJ, USA), using the Illumina RNA platform with PolyA selection sequencing protocol, 20 million reads per sample.

Since there is no *A. vladimiri* genome reference available, a reference transcriptome was built *de novo* from AnvRV^−^ wasps (*n* = 5). *De novo* assembly was done using Trinity rnaseq v2.14.0 (42). Trinity assembly quality was evaluated using BUSCO 5.4.2 (43). Differential expression analysis was achieved by quantification and normalization of the assembled reads (SALMON v1.9.0 [44]), followed by statistical analysis and visualization performed using DESeq2 (45). Trinotate v3.2.2c (46) was used to annotate the transcripts, ShinyGo (47) was used for GSEA, and Blastx (48) was used to annotate the differentially expressed genes. To test for differentially expressed gene sets, gene sets defined based on prior biological knowledge were selected, and a false discovery rate (FDR) <0.01 was used as a threshold.

### Quantitation of selected differentially expressed genes by qPCR

To test the hypothesis that AnvRV alters *A. vladimiri’s* venom composition, differentially expressed (DE) genes (adjusted *P* value < 0.0001) were compared to genes found in the venom of other hymenopteran species, mainly parasitoid wasps. Out of the highly DE genes identified by the transcriptome analyses and found in parasitoid wasps’ venom production pathway (Supplemental material), four genes, which potentially have DE genes between ovaries and venom gland, were selected for further analysis and validation of the transcriptome results: Omega conotoxin, Nasonin, NF-κB inhibitor, and venom carboxylase. Omega conotoxin is a well-studied protein that inhibits N-type voltage-dependent calcium channels (49) and is likely part of the venom fluid. Nasonin is a gene found so far only in the venom of the parasitoid *Nasonia vitripennis* (Hymenoptera: Pteromalidae) and was found to inhibit the host’s melanization response (50). NF-κB is an immune response transcription factor that governs the transcription of two major immune response pathways: Toll and Imd (51); NF-κB inhibitor inhibits this process. Carboxylase is a metabolic gene found in the venom of *Chouioia cunea* (33). To further test expression specificity, AnvRV^+^ and AnvRV^−^ *A. vladimiri* female wasps were dissected in ML buffer (from the RNA extraction kit) and stored at −20°C until RNA extraction. Total RNA of the maternal injection system and ovaries (pools of 10 individuals each), as well as three pools of five whole wasps, was separately extracted using the Macherey-Nagel kit according to the manufacturer’s manual and eluted in a 30 µL final elution volume.

RNA concentrations were measured using Qubit, and samples’ concentrations were normalized before cDNA synthesis (Supplemental material). RNA samples were reverse-transcribed to cDNA using the RT-PCRbio kit (PCR Biosystems). Specific primers for qPCR were designed for the selected genes, in addition to AnvRV (Supplemental material). Fast SYBR Green Master Mix (Thermo Fisher) was used according to protocol. The programs were set as follows: enzyme activation at 95°C for 30 s, followed by 40 cycles with denaturation at 95°C for 5 s, annealing and extension at 60°C for 30 s, and a melting curve analysis. mRNA expression levels and virus quantification were normalized according to the reference gene heterogeneous nuclear ribonucleoprotein K.

To assess differences in gene expression (measured as fold change) across treatment groups and tissue types, we performed one-way and two-way analyses of variance, followed by post hoc multiple comparisons using Tukey’s honest significant difference test.

### Screening of *Anagyrus vladimiri* viruses by data mining

To test if the three *A. vladimiri-*associated viruses could be found in other wasp species within the family Chalcidoidae or within mealybugs, their homologs were searched in the majority of the RNAseq data sets of these host taxa available in the NCBI SRA database as of November 2023. This method has the potential to augment the discovery of RNA virus sequences beyond what was previously achieved in large meta-analyses of RNAseq data (34, 52, 53), because, unlike meta-analyses that rely solely on homology with the RdRP sequences, this approach involves using the entire viral sequence assembly of the three novel viruses.

To reduce computing resource requirements, a filtering of the RNAseq data sets was obtained; PacBio data sets were removed due to technical difficulties, and only a few Illumina data sets were kept for each wasp/mealybug organism, preferably sequenced from the whole body of females (if this information was indicated in the metadata). A total of 121 SRA data sets originating from 82 species within 19 families of Chalcidoidea were used in the analysis (see Supplementary material RNA datamining table). Out of the 82 species, five belonged to the Encyrtidae family (*Aenasius bambawalei, Ooencyrtus telenomicida, Metaphycus flavus, Leptomastix dactylopii, Copidosoma floridanum*). While only *L. dactylopii* parasitizes mealybugs (like *A. vladimiri*), the four other Encyrtid species parasitize either soft scale insects, stink bugs, or moths (Table 1).

The SRA list of Pseudococcidae totaled 134 data sets originating from 84 species (SRA accession numbers and metadata are available in the supplementary material RNA datamining table). For positive controls, we used the SRA data sets of (i) our previous study on *A. vladimiri* (20), and (ii) a study that identified very similar viruses in vine mealybugs in Mexico (37).

The SRA data sets were downloaded using the SRA toolkit (version 3.0.8). As a feasibility test, we first screened only the SRA data sets originating from Encyrtidae wasps, using a DIAMOND-BLAST quick search (see Results). However, due to technical and computing resources limitations, the full screen was done using SALMON (44). Adapter removal and trimming were performed using trim_galore version 0.5.0 (https://github.com/FelixKrueger/TrimGalore) with default parameters. For paired-end reads, the read_qc module from the metaWRAP version 1.3.2 (54) pipeline was used as a wrapper for trim_galore. High-quality clean reads were used to quantify *A. vladimiri* viruses using Salmon version 1.9.0 (44) in alignment-free mode. The SALMON strictness parameter --minScoreFraction was adjusted to 0.3 to improve the sensitivity of the screening for detecting viral sequences with low similarity to *A. vladimiri* viruses.

A minimum of 10 reads was required to consider a positive result for a virus in an SRA data set. Analysis was performed using the R Environment for Statistical Computing (R Core Team [2024] version 4.2.2). To obtain full viral sequences from SRA data sets in which viral transcripts were found, the assembled contigs were downloaded from the Transcriptome Shotgun Assembly (TSA) Database. If this data were not available, a *de novo* assembly was done on the SRA raw reads, using Trinity v2.14.0 (42).

To assess the similarities (phylogenetic relationship) between the viral sequences found, the contigs were compared specifically to the *A. vladimiri* viruses using tblastx and blastn. Additionally, a phylogenetic tree was reconstructed using the RdRP coding sequences, as detailed in reference 20. Briefly, RdRP sequences (~300 amino acids long) were derived from translated open reading frames by annotating them using the conserved domain database tool. A few representative sequences from the relevant virus families were added. A multiple sequence alignment was done using ClustalO, and a maximum-likelihood tree was reconstructed.

### Viruses’ prevalence in field-collected wasps and mealybugs

To assess the prevalence of the three viruses of *A. vladimiri* in the field, mealybugs were collected from a vineyard near Dalton, Israel (33.017336, 35.498344) in September 2023, and from citrus trees near Ramat Yishay, Israel (32.694920, 35.155145) in June 2024. The collected mealybugs were kept in the lab in cages until parasitoid emergence (~2 weeks). The parasitoids and their hosts were collected into sterile microcentrifuge tubes and kept in −80°C till processed.

Molecular identification of the mealybugs was done using molecular barcodes as described by Daane et al. (55), morphological identification of *A. vladimiri* was established by GW and YI, and that of the other parasitoid species by Dr. Miriam Kishinevsky. All parasitoids’ identity was further tested molecularly by PCR amplification of the 18S gene using in-house designed primers (fragment size 900 bp, 18S-F AGATACCGCCCTAGTTCTAACC; 18S-R GATCCTTCTGCAGGTTCACC), and the resulting sequences were compared with known sequences in the databases, using BLASTn search. Parasitoids and mealybugs collected in the field were screened for the presence of AnvRV, *Anagyrus vladimiri* Dicistrovirus, and *Anagyrus vladimiri* Iflavirus according to the protocol described in Izraeli et al. (20). Host mealybugs were collected in bulk and maintained in emergence cages under controlled laboratory conditions to facilitate the recovery of adult wasps. Because the study focused on the viral status of the emerging parasitoid population, the initial host density was not quantified, and the percentage of parasitization was not determined.

## Data Availability

Transcriptome data are available at Bioproject accession PRJNA1033425, SRR33332457–SRR33332466, and in the supplemental material.

## SUPPLEMENTAL MATERIAL

Fig. S1 spectrum.01636-25-s0001.tif TEM of AnvRV+ *Anagyrus vladimiri* ovaries.

Fig. S2 spectrum.01636-25-s0002.tif TEM of AnvRV+ *Anagyrus vladimiri* venom system.

Supplemental material spectrum.01636-25-s0003.docx Supplemental figure legends.

Supplemental tables spectrum.01636-25-s0004.xlsx Tables S1 to S6.

ASM does not own the copyrights to Supplemental Material that may be linked to, or accessed through, an article. The authors have granted ASM a non-exclusive, world-wide license to publish the Supplemental Material files. Please contact the corresponding author directly for reuse.
