# Supplementary figures and images for: AnvRV virus in the parasitoid wasp Anagyrus vladimiri: localization, effect on gene expression, and prevalence
Source: Microbiol Spectr. 2026 May 26;14(7):e01636-25. doi: 10.1128/spectrum.01636-25 (PMC13340311; doi:10.1128/spectrum.01636-25)

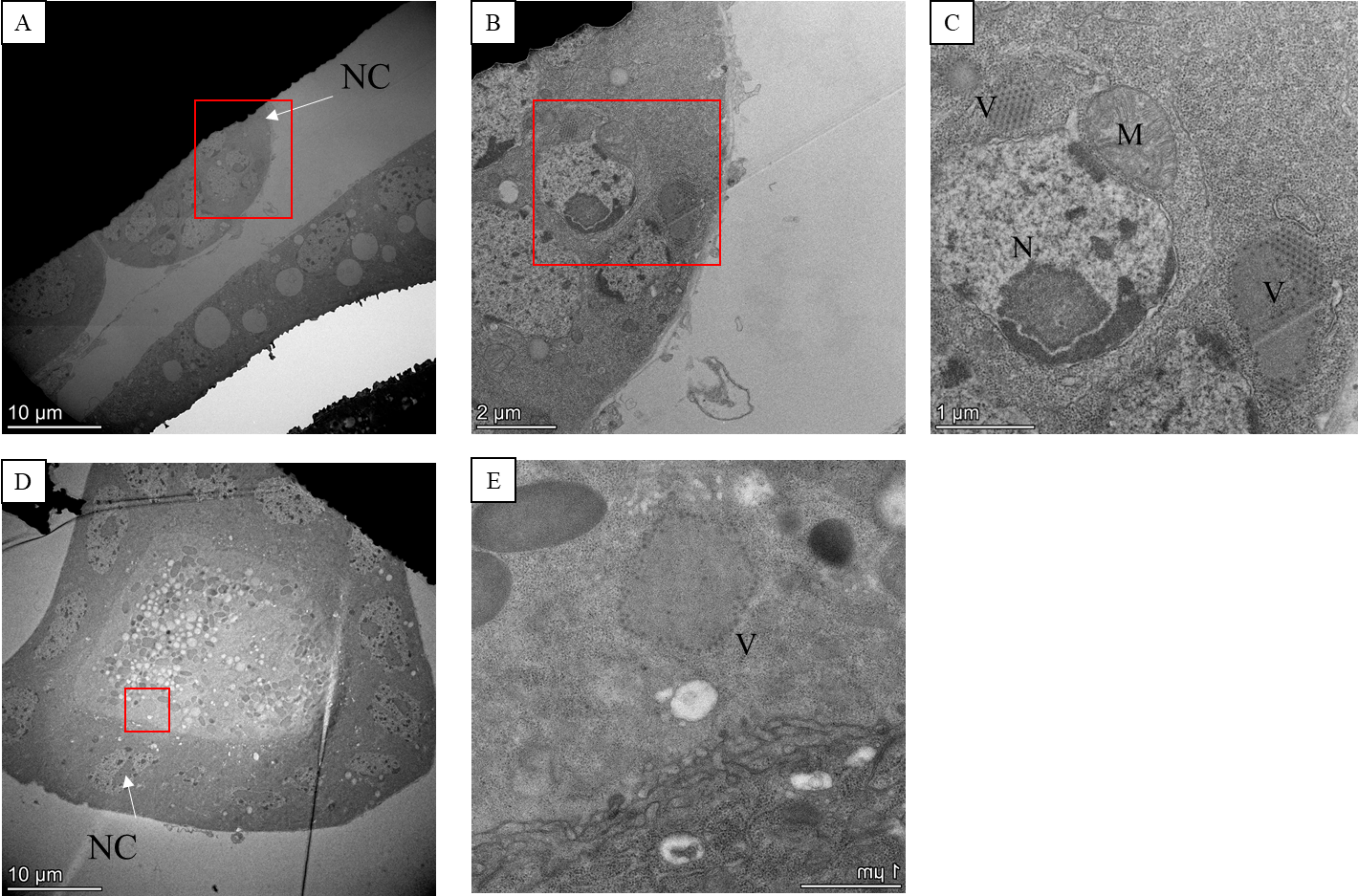

Supplement: Fig. S1 — TEM of AnvRV+ Anagyrus vladimiri ovaries. [file spectrum.01636-25-s0001.tif]

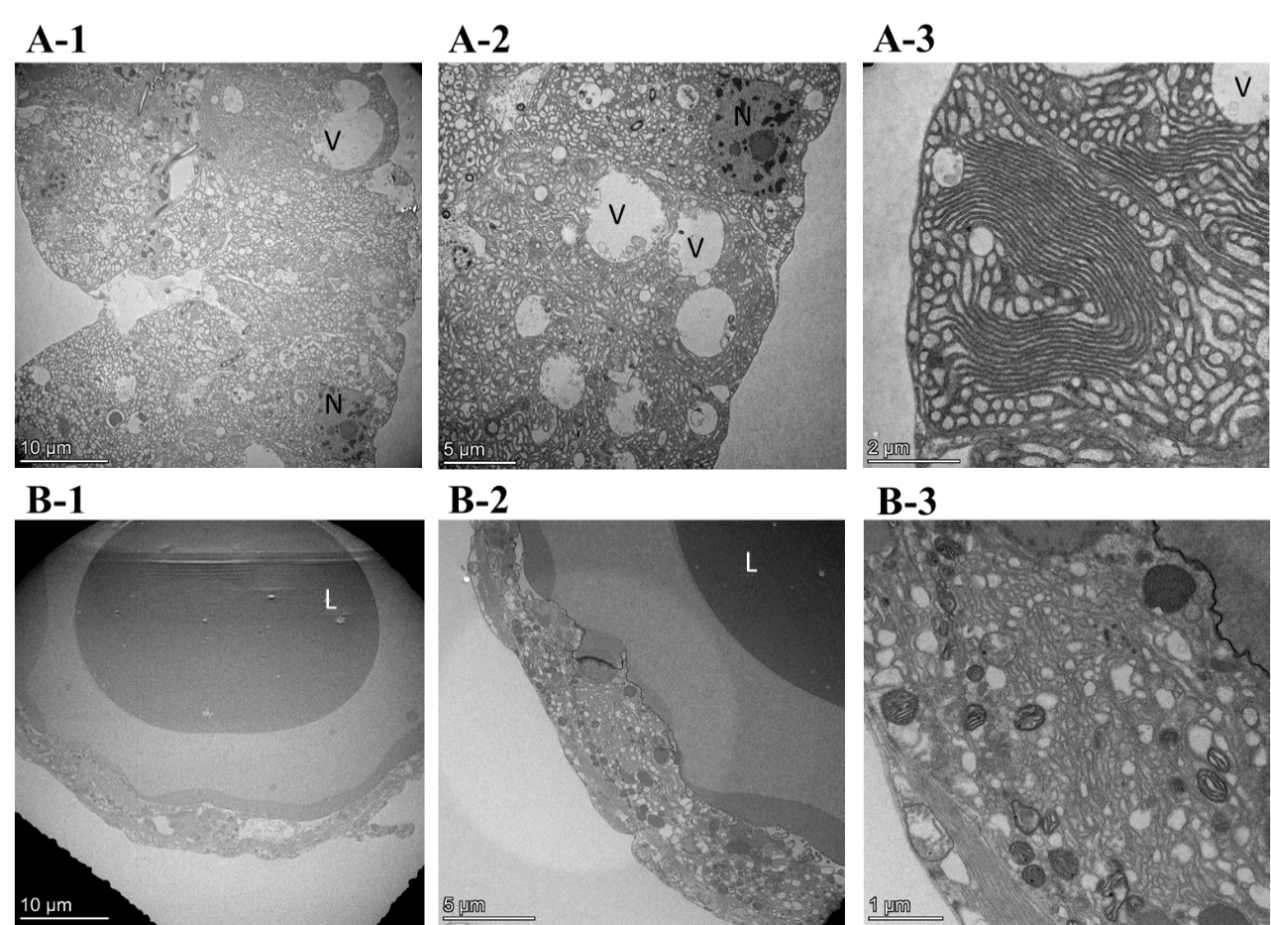

Supplement: Fig. S2 — TEM of AnvRV+ Anagyrus vladimiri venom system. [file spectrum.01636-25-s0002.tif]
