# Supplementary material for: AnvRV virus in the parasitoid wasp Anagyrus vladimiri: localization, effect on gene expression, and prevalence
Source: Microbiol Spectr. 2026 May 26;14(7):e01636-25. doi: 10.1128/spectrum.01636-25 (PMC13340311; doi:10.1128/spectrum.01636-25)
Supplement: Supplemental material — Supplemental figure legends. [file spectrum.01636-25-s0003.docx]

Supplemental figure legends

Figure S1. TEM of AnvRV^+^ *A*nagyrus *vladimiri* ovaries. (A) Nurse cells of AnvRV^+^ *A*. *vladimiri*; (B) enlargemnt of "A"; (C) enlargement of "B" ; (D) Young oocyte with nurse cells around; (E) Enlargement of "D"; NC – Nurse cells, M – Mitochondria, N- Nucleus, V – Virus particles, red squares represent the enlargement area;

Fig. S2. TEM of AnvRV^+^ *A*nagyrus *vladimiri* venom system. No virus could be seen in both in the venom gland (A) and in the venom resrvoir (B). Venom (V); Nucleus (N); Lumen (L).
